# Clinical presentation and transmission of postnatal cytomegalovirus infection in preterm infants

**DOI:** 10.3389/fped.2022.1022869

**Published:** 2022-11-21

**Authors:** Jun Eon Lee, Yea Seul Han, Tae-Jung Sung, Dong Hyun Kim, Byung Ok Kwak

**Affiliations:** ^1^ Department of Pediatrics, Hallym University Kangnam Sacred Heart Hospital, Seoul, South Korea; ^2^ Department of Pediatrics, Inha University School of Medicine, Incheon, South Korea

**Keywords:** cytomegalovirus, premature infant, human milk, transmission, viral load

## Abstract

**Background:**

Preterm infants are at greater risk of developing postnatal cytomegalovirus (CMV) infection with serious symptoms. Breast milk is the main route of CMV transmission in populations with a high seroprevalence.

**Objectives:**

This study aimed to investigate the clinical presentation and transmission of postnatal CMV (pCMV) infection *via* breast milk in preterm infants under the specific setting of our neonatal intensive care unit (NICU).

**Methods:**

The medical records of 147 preterm infants were reviewed retrospectively, and their clinical characteristics and outcomes were analyzed. Breast milk and infant urine samples were collected every two weeks until discharge, and the kinetics of CMV loads were evaluated using a polymerase chain reaction assay.

**Results:**

Seventeen infants (11.6%) were diagnosed with pCMV infection during the study period. In comparison between the pCMV and control groups, the mean birth weight was significantly lower in the pCMV group than in the control group (1084.1 ± 404.8 g vs. 1362.5 ± 553.8 g, *P* = 0.047). Four (23.5%) patients had leukocytopenia, six (35.3%) had neutropenia, three (17.6%) had thrombocytopenia, and two (11.8%) had hyperbilirubinemia in the pCMV group. Five patients were treated with antiviral agents, and their CMV load in the urine decreased after treatment. CMV loads peaked at 3–5 weeks in breast milk, whereas they peaked at 8–12 weeks of postnatal age in infants' urine. A comparison between the median CMV load in breast milk from the pCMV and control groups revealed a significant difference (*P* = 0.043).

**Conclusion:**

Most preterm infants with pCMV infection present a favorable clinical course and outcomes. A high CMV viral load in breast milk is associated with transmission. Further studies are warranted to prevent transmission and severe pCMV infections in preterm infants.

## Introduction

Cytomegalovirus (CMV) is a common cause of intrauterine and perinatal infection worldwide. Perinatal infections occur during exposure to genital secretions at birth or *via* breast milk. Breastfeeding is the most common route of CMV transmission from seropositive mothers to their babies postnatally (1–4). Most CMV seropositive mothers become reactivated during lactation up to 96% and excrete the virus in breast milk without clinical or laboratory signs of systemic infection (5–13). CMV transmission rate through breast milk is 58%–69% in term infants, 5.7%–58.6% in preterm infants, and 38% in preterm infants with birth weights of <1,500 g or GA <32 weeks (2, 8, 14, 15). Postnatal CMV (pCMV) infection risk may be greater in populations with a high prevalence of seropositivity (16, 17). The maternal CMV-IgG positivity rate was reported to be in the range 52%–97% (8). In Korea, ≥80% of women have CMV antibodies by childbearing age, and the CMV transmission rate from mothers to preterm infants through breast milk is estimated to be high (18, 19).

pCMV infections are usually asymptomatic without long-term sequelae or hearing problems. Preterm infants may have a higher risk of pCMV acquisition *via* breast milk because of immature immunity and insufficient maternal antibodies. Hence, they may present serious clinical manifestations such as neutropenia, thrombocytopenia, pneumonitis, hepatitis, hepatosplenomegaly, neonatal jaundice, intestinal manifestations, and sepsis-like symptoms (SLS) (4, 6–8, 15, 20).

This study aimed to investigate the incidence and clinical presentation of pCMV infection *via* breast milk and assess the kinetics of CMV viral loads in breast milk and urine among preterm infants during their hospitalization in the specific setting of our neonatal intensive care unit (NICU).

## Materials and methods

Medical records of 182 preterm infants born at ≤34 weeks of gestational age who were admitted to the neonatal intensive care unit (NICU) of Hallym University Kangnam Sacred Heart Hospital between September 2016 and September 2020 were reviewed retrospectively. Infants who were transferred to other hospitals (*N* = 13), who died within three weeks after birth (*N* = 5), or whose research was terminated before 36 weeks of corrected age (*N* = 15) were excluded. Infants with congenital CMV infection in which CMV DNA was isolated from their urine within the first three weeks of life (*N* = 2) were excluded as well. Consequently, 147 preterm infants were included in this study.

Mothers were not routinely screened for CMV serostatus in our hospital. Owing to the high seroprevalence of 95.8% in Korean women of childbearing age (17), CMV screening tests have been routinely performed during admission to our NICU. Initial CMV polymerase chain reaction (PCR) was performed on infants' urine and their mother's breast milk within the first 14 days of life. For the surveillance of pCMV infection, CMV PCR of urine samples was repeated if the infant was clinically suspected of CMV infection or every two weeks until discharge.

All infants were fed their mothers' raw breast milk which was refrigerated at 4 °C for ≤24 h, or freeze-thawed breast milk until the breast milk was CMV PCR positive. Formula milk was used to supplement the insufficient breast milk. If CMV, which was tested weekly with PCR, was isolated from the breast milk, the infant was fed formula milk instead for the rest of the hospitalization period. Prior to testing breast milk samples were stored at 4 °C for up to 24 h or −20 °C for 2 weeks of collection. The CMV load in the breast milk and infant urine was measured using quantitative real-time PCR (Real-Q CMV Quantification Kit; Applied BioSewoom, Seoul, South Korea). The detection limit of this assay is approximately 100 copies/ml.

The incidence of pCMV infection was assessed throughout the study period. Clinical characteristics including gestational age, birth weight, sex, mode of delivery, Apgar score at 1 and 5 min, small for gestational age (SGA), intrauterine growth restriction (IUGR), maternal age, pregnancy-induced hypertension, diabetes mellitus, chorioamnionitis, antenatal steroid use, and the number of transfusions were analyzed. Outcome measures included intraventricular hemorrhage (IVH), ventriculomegaly, periventricular leukomalacia (PVL), respiratory distress syndrome (RDS), bronchopulmonary dysplasia (BPD) (21), necrotizing enterocolitis (NEC) (22), sepsis, retinopathy of prematurity (ROP), and sensorineural hearing loss (SNHL). Abnormal laboratory findings including leukopenia (white blood cell counts <4,000/mm^3^), neutropenia (absolute neutrophil counts <1,000/mm^3^), thrombocytopenia (platelet level <100,000/mm^3^), elevated liver enzymes (aspartate aminotransferase or alanine aminotransferase level >60 IU/L) or hyperbilirubinemia (total bilirubin level >10 mg/dL or direct bilirubin level >2 mg/dL) were assessed and compared between pCMV infection and non-infection (control) groups. The control infants were all the uninfected infants admitted to the NICU in the same period, based on the negative viruria during the hospitalization. Case and control cases were not matched by gestational age and/or birth weight.

The study protocol was approved by the Institutional Review Board of Hallym University Kangnam Sacred Heart Hospital (IRB No. 2021-10-019), and the requirements for informed consent were waived for this retrospective chart review. Data are expressed as the mean ± standard deviation, median [95% confidence interval (CI)], or number (frequency). Comparisons between continuous variables were performed using the independent *t*-test or Mann–Whitney *U* test, and comparisons between categorical variables were performed using the chi-square test or Fisher's exact test. Statistical significance was set at *P *< 0.05. Statistical analyses were performed using IBM SPSS Statistics version 24 software (IBM Corp., Armonk, NY, USA).

## Results

**Figure 1:**
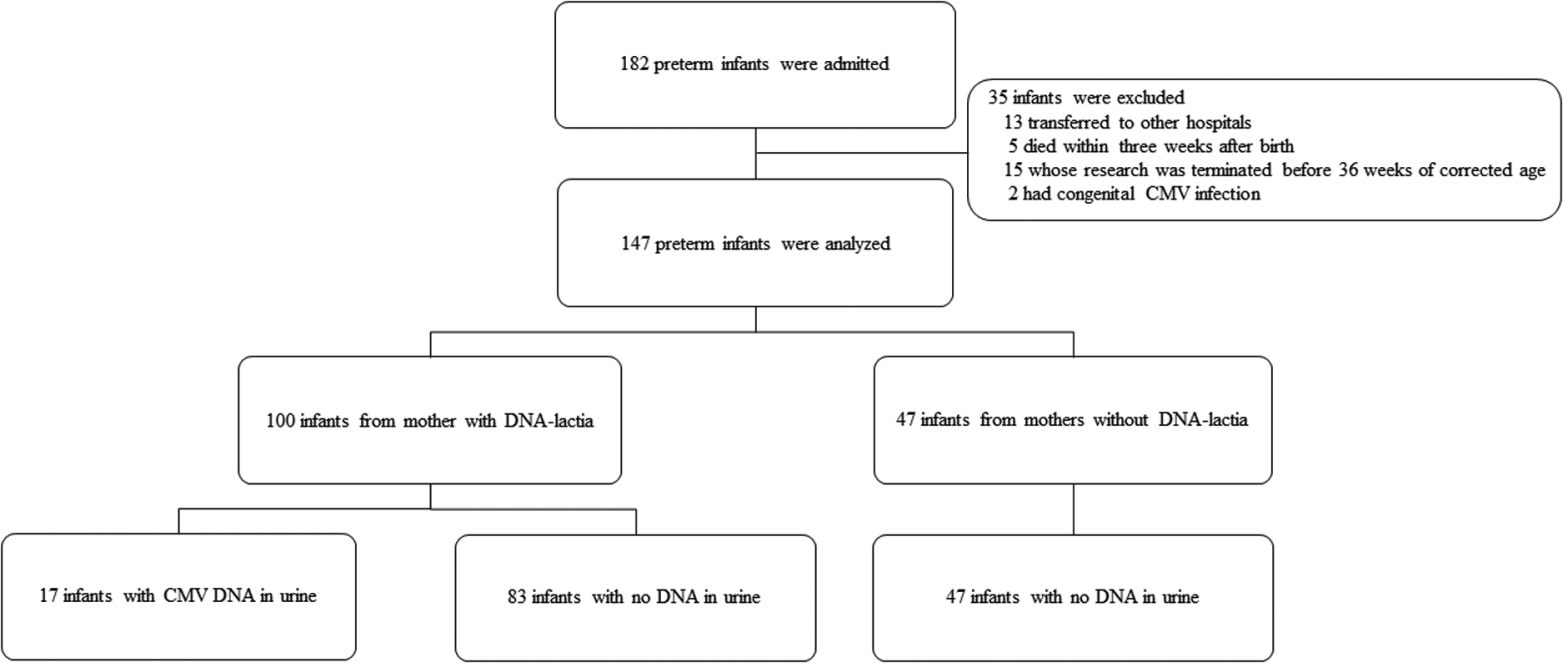
Flow diagram showing the enrollment and laboratory testing of the study population. CMV, cytomegalovirus.

The enrollment and laboratory testing of the study population is presented in Figure 1. During the study period, pCMV infection was diagnosed in 11.6% (17/147) of preterm infants. The median time to the first detection of CMV DNA in the urine was 36 days (range, 19–64 days). Of the 100 infants from mothers with CMV shedding into breast milk, 17% (17/100) were identified as having pCMV infection. The demographic characteristics of the study groups are shown in Table 1. In comparison between the pCMV and control groups, the mean birth weight was significantly lower in the pCMV group than in the control group (1084.1 ± 404.8 g vs. 1362.5 ± 553.8 g, *P* = 0.047). Maternal chorioamnionitis was more common in the pCMV group than in the control group (23.5% vs. 6.2%, *P* = 0.046). Other variables, including, mean gestational age and Apgar score, were not significantly different between the two groups. The median hospitalization duration was 92 and 59 days (*P *= 0.005), and the median number of urine samples obtained from each infant was 7 and 4 (*P *< 0.01) in the pCMV and control groups, respectively. All 17 breast milk samples from the pCMV group were CMV PCR-positive. The median duration of breastfeeding was 36 days and 59 days in the pCMV and the control groups, respectively (*P *< 0.01).

**Table 1:** Demographic findings of the postnatal cytomegalovirus infection and control groups.

	pCMV (*N* = 17)	Control (*N* = 130)	*P*-Value
Gestational age (week)	27.4 ± 2.5	28.9 ± 3.2	0.065
Birth weight (g)	1084.1 ± 404.8	1362.5 ± 553.8	0.047
Cesarean section	12 (70.6)	101 (77.7)	0.514
APGAR score at 1 min	3.2 ± 2.2	4.1 ± 2.2	0.143
APGAR score at 5 min	4.9 ± 2.9	6.2 ± 2.2	0.100
Small for gestational age	0 (0)	8 (6.2)	0.293
Intrauterine growth restriction	1 (5.9)	7 (5.4)	0.933
Maternal age (year)	34.6 ± 3.0	33.2 ± 4.4	0.193
Pregnancy induced hypertension	0 (0)	15 (11.5)	0.309
Gestational diabetes mellitus	0 (0)	16 (12.3)	0.285
Maternal chorioamnionitis	4 (23.5)	8 (6.2)	0.046
Antenatal steroid treatment	17 (100)	112 (86.2)	0.132
Transfusion	8 (47.1)	46 (35.4)	0.348
No. of transfusion	2.5 ± 3.6	1.4 ± 2.2	0.078

Data are presented as the mean ± standard deviaion or number (%).

pCMV, postnatal cytomegalovirus infection.

The clinical course and adverse outcomes were compared between the two groups (Table 2). The incidences of RDS (94.1% vs. 60.8%, *P* = 0.007) and BPD (64.7% vs. 39.2%, *P *= 0.045) were significantly higher using univariate analysis in the pCMV group than was in the control group. SNHL occurred in one (5.9%) infant in the pCMV group. The infant was born at 24 weeks of gestational age with a birth weight of 600 g, and was exposed to ototoxic drugs during hospitalization. Among 17 infants in the pCMV group, four patients (23.5%) had leukocytopenia, six (35.3%) had neutropenia, three (17.6%) had thrombocytopenia, and two (11.8%) had hyperbilirubinemia. Neutropenia (35.3% vs. 3.8%, *P *< 0.01) and elevated aspartate aminotransferase (41.2% vs. 18.5%, *P* = 0.031) were observed more in the pCMV group than in the control group. Other adverse outcomes such as IVH, ventriculomegaly, PVL, sepsis, and ROP were not significantly different between the two groups. None of the groups showed calcification of the brain, hepatosplenomegaly, or NEC. In multivariate logistic regression after adjustment for gestational age and birth weight, only neutropenia was significantly different between the two groups (*P *< 0.01).

**Table 2:** Clinical course and outcomes of the postnatal cytomegalovirus infection and control groups.

	pCMV (*N* = 17)	Control (*N* = 130)	*P*-Value	*P*-Value*
Intraventricular hemorrhage (≥grade 2)	5 (23.1)	30 (29.4)	0.564	0.175
Ventriculomegaly	2 (11.8)	7 (5.4)	0.302	0.851
Periventricular leukomalacia	1 (5.9)	6 (4.6)	0.818	0.999
Respiratory distress syndrome	16 (94.1)	79 (60.8)	0.007	0.060
Bronchopulmonary dysplasia	11 (64.7)	51 (39.2)	0.045	0.108
Sepsis	2 (11.8)	27 (20.8)	0.380	0.178
Retinopathy of prematurity	6 (35.3)	23 (17.7)	0.086	0.706
Sensorineural hearing loss	1 (5.9)	0 (0)	0.006	0.999
**Laboratory findings**				
Leukopenia	4 (23.5)	29 (22.3)	0.910	0.093
Neutropenia	6 (35.3)	5 (3.8)	<0.01	<0.01
Thrombocytopenia	3 (17.6)	38 (29.2)	0.317	0.520
AST elevation	7 (41.2)	24 (18.5)	0.031	0.192
ALT elevation	2 (11.8)	4 (3.1)	0.089	0.230
Hyperbilirubinemia	2 (11.8)	6 (4.6)	0.222	0.955

Data are presented as the mean ± standard deviation or number (%).

AST, aspartate aminotransferase; ALT, alanine aminotransferase; pCMV, postnatal cytomegalovirus infection.

* *P*-values are calculated after adjustment for gestational age and birth weight.

In the pCMV group, five infants (29.4%) were administered with ganciclovir, an antiviral agent, and 12 infants were not. When comparing the clinical characteristics of the treated and untreated patients, the median gestational age (25.2 weeks vs. 28.5 weeks, *P* = 0.002) and birth weight (700.0 g vs. 1235.0 g, *P *= 0.006) were lower in the treated patients. All five patients with antiviral treatment were born at ≤26 weeks of gestational age, had a birth weight of ≤1,000 g, and developed symptomatic pCMV infection, such as apnea with bradycardia, neutropenia, thrombocytopenia, elevated liver enzymes, hyperbilirubinemia, or enteral feeding intolerance; SNHL was observed in one patient performed at 4 months of age (Table 3). CMV DNA was first detected in the urine at a median of 48 postnatal days (ranges, 36–53 postnatal days) among the five infants. CMV PCR of blood samples was performed in two infants, and confirmed to be positive. In three infants, CMV IgM was positive. Antiviral treatment was initiated at a mean of 74 postnatal days (ranges, 47–87 postnatal days), and no adverse drug reactions were reported. Ganciclovir 6 mg/kg/dose was administered intravenously every 12 h, and the duration of treatment was 6 weeks for three infants and 3 weeks for two infants. The CMV load in the breast milk and in the urine of the five preterm infants is shown in Supplementary Figure S1. Viral loads decreased after treatment in all patients.

**Table 3:** Clinical characteristics of the patients with ganciclovir treatment.

	GA (week)	BW (g)	Sex	Mode of Delivery	IVH (≥Gr3)	Ventriculomegaly	PVL	RDS	BPD	ROP	SNHL	Neutropenia	Thrombocytopenia	AST/ALT elevation
P1	23 + 3	530	F	NSVD	−	+	−	+	+	+	−	–	+	+
P2	26 + 0	900	M	NSVD	−	−	−	+	+	+	−	+	−	−
P3	24 + 1	600	M	C/S	−	+	+	+	+	+	+	−	−	+
P4	25 + 1	700	M	C/S	+	+	−	+	+	+	−	+	−	+
P5	25 + 2	780	F	C/S	−	−	−	+	+	+	−	+	−	−

ALT, alanine aminotransferase; AST, aspartate aminotransferase; BPD, bronchopulmonary dysplasia; C/S, cesarean section; GA, gestational age; Gr, grade; IVH, intraventricular hemorrhage; NSVD, normal spontaneous vaginal delivery; PVL, periventricular leukomalacia; RDS, respiratory distress syndrome; ROP, retinopathy of prematurity; SNHL, sensorineural hearing loss.

**Figure 2:**
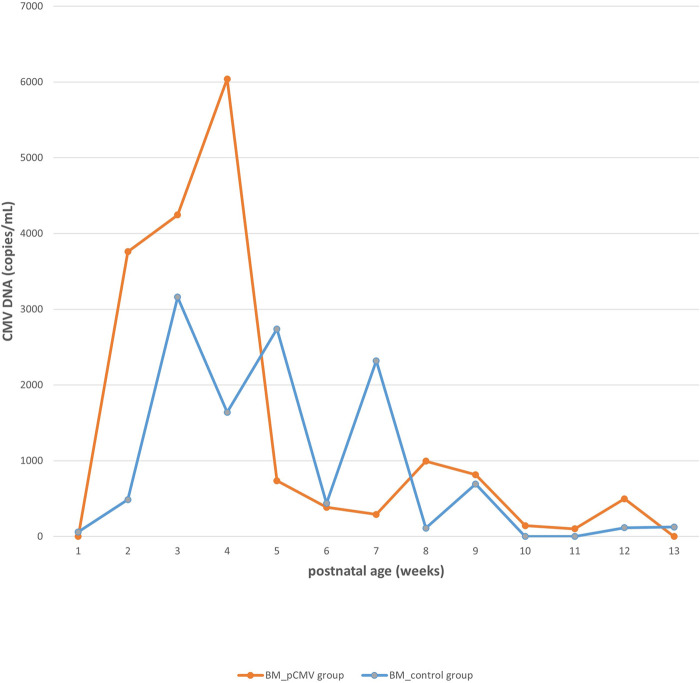
The kinetics of cytomegalovirus loads in breast milk. Orange and blue lines indicate the median copy numbers of CMV DNA in the breast milk of pCMV and control groups. CMV, cytomegalovirus; BM, breast milk; pCMV, postnatal cytomegalovirus.

**Figure 3:**
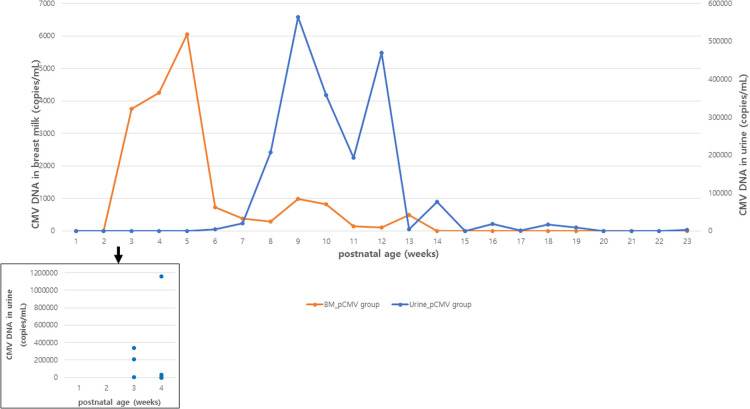
Median CMV load in breast milk and urine samples in infants with postnatal CMV infection. Orange and blue lines indicate the median copy numbers of CMV DNA in breast milk and urine. Blue dots indicate the copy numbers of CMV DNA in urine of infants with CMV PCR positive urine at 1 to 4 postnatal weeks. BM, breast milk; CMV, cytomegalovirus.

The CMV viral load in breast milk was determined, and its association with the transmission was analyzed. The viral loads increased until they peaked at 3–5 postnatal weeks, after which they decreased in both groups (Figure 2). The median viral load in the breast milk from the pCMV group was significantly higher than that from the control group (*P *= 0.043). In the pCMV group, the highest values of CMV DNA copies were detected in breast milk between 3 and 5 weeks of postnatal age and 8–12 weeks after birth in infant urine (Figure 3).

## Discussion

In our study, the incidence of pCMV infection was 11.6% (95% CI, 6.7%–18.5%) in preterm infants, and 17% (95% CI, 9.9%–27.2%) in infants of mothers with CMV shedding into breast milk. Breast milk was the main route of transmission. Most infants with pCMV infection showed favorable clinical outcomes, even though five infants were treated with antiviral agents. CMV viral loads peaked at 3–5 and 8–12 weeks of postnatal age in breast milk and infant urine samples, respectively. Significantly higher median CMV loads in breast milk were observed in the pCMV group than in the control group.

In a meta-analysis including 2,502 infants from 19 studies, the pCMV infection rate was 16.5% in infants from seropositive mothers, and 20.7% in infants fed CMV-positive breast milk (5). Previous studies have shown that extremely preterm infants are at high risk of developing serious symptoms and sequelae following pCMV infection (7, 16, 23–25). This might be explained by the absence of prenatally acquired anti-CMV antibodies during early gestational age, in combination with the presence of CMV in breast milk, which increases the risk of pCMV infection through breast milk among extremely preterm infants (16). This is consistent with our study showing that lower birth weight and gestational age was observed in the pCMV group than in the control group. Although five infants born at ≤26 weeks of gestational age were treated with antiviral agents, most infants had nonspecific manifestations of CMV infection, and their symptoms were not severe. In multivariate analysis, the only significant difference in the pCMV group compared to the control group was neutropenia, which is in line with a previous study (26). SNHL was observed in one infant born at 24 weeks of gestational age with a birth weight of 600 g, who was exposed to ototoxic drugs during hospitalization. SNHL might not be clearly related to pCMV infection. The incidence of pCMV infection can be affected by ethnicity, seroprevalence of the mothers, time of the screening test, and gestational age and birth weight of the participants. For surveillance of pCMV infection, CMV PCR of urine samples was routinely performed every two weeks until discharge; thus, it could not be underestimated. However, in some infants, breastfeeding was discontinued early after DNA-lactia was detected, thus, the incidence of pCMV infection might be lower. In addition, it is presumed that freeze-thawing or pasteurization of breast milk might lead to different results regarding the incidence of breast milk-transmitted pCMV infection.

In our study, using multivariate logistic regression, the incidence rates of RDS and BPD were not higher in the pCMV group than in the control group. Previous studies reported that pCMV infection was associated with an increased risk of BPD (16, 27), but this association was not found in this study. The higher incidence of RDS was mediated by the lower birth weight and the higher incidence of chorioamnionitis in the pCMV group than in the control group. There is no consensus guideline on the management of pCMV infection in preterm infants or the use of antiviral treatment in severely symptomatic cases (28, 29). Moreover, data on the long-term outcomes after pCMV infection in preterm infants are limited and controversial. Kelly et al. (27) demonstrated that pCMV infection was associated with an increased risk of BPD in VLBWIs at 36 weeks of postnatal age. Some studies have reported poor neurocognitive outcomes in adolescents born preterm with pCMV infection. In contrast, other studies have shown that preterm infants might not have an increased risk for neurological sequelae, and the differences in neurocognitive outcome could only be detected in higher ages, >6 years (2, 8, 12, 16, 18, 30, 31). Further research is required to understand the disease burden and establish guidelines for the prevention and management of pCMV infection in preterm infants from CMV-seropositive mothers.

Most CMV DNA in breast milk begins to be detected two weeks after delivery and increases until 4–6 weeks after delivery. CMV detection in the urine of newborns with pCMV is between 4 and 12 weeks, with an average of 6–8 weeks (3, 7, 14, 32, 33). This means that CMV DNA is not detected immediately in the urine of newborns exposed to CMV- PCR-positive breast milk, and mother-to-infant transmission occurs several weeks later (3). In our study, CMV viral loads in breast milk increased until they peaked at 3–5 postnatal weeks, and CMV DNA in most infant urine was detectable after 6 weeks of age. Furthermore, a higher viral load in breast milk was observed in the pCMV group than in the control group. This is consistent with previous studies showing that the early onset of virolactia and high viral load in breast milk are risk factors for CMV transmission (7, 25). In a Japanese and an Italian study, CMV DNA was detectable in most breast milk samples 2–3 weeks after delivery and increased until they peaked at 4–6 weeks (3, 9).

In our study, the median time to the first detection of CMV DNA in the urine was 36 days, but CMV DNA was first detected in the urine at 3 postnatal weeks in three infants. This supports that pCMV infection *via* breast milk can occur early (<3 weeks) after birth in a small (3 of 17) number of preterm infants, hence, the confirmation test to diagnose congenital CMV infection should be performed within 3 weeks, ideally within 2 weeks of birth in highly seropositive populations, as suggested in a previous study (34).

The maximum CMV load in breast milk was higher in uninfected infants than in CMV-infected infants (3). On the other hand, higher median CMV load and longer CMV DNA detection time in breast milk were observed in infected infants compared to uninfected infants in a Taiwanese study (33). In a German study, CMV DNA or infectious virus was detectable in the infant's urine a mean of 42 days after the first exposure to CMV-positive breast milk, with a mean incubation period of 47 days (7). The early appearance of both CMV DNA and infectious virus in milk whey or milk cells were risk factors for transmission (24). More detailed studies are needed to elucidate the kinetics of CMV reactivation in breast milk and identify the risk factors for transmission.

This study has several limitations. First, owing to the small number of infants with symptomatic pCMV infection, we could not perform further statistical analyses to determine the risk factors for acquiring the disease. Another limitation of this study is the inclusion criteria being different from previous studies. Although preterm infants born at <34 weeks of gestational age were included, the mean gestational age was 27.4 ± 2.5 weeks (ranges, 23–32 weeks), and there was only one infant born at 32 weeks of gestational age in the pCMV group. Therefore, it is considered to be possible to compare the results of our study with those of other studies including preterm infants with gestational age <32 weeks or birth weight <1,500 g. Third, the specific management of stopping breastfeeding when DNA-lactia was found may have affected the incidence of pCMV infection. It could not be verified whether infants breastfed for longer had a higher risk of pCMV infection due to this management. Forth, long-term outcome data on the effects of pCMV infection, such as neurodevelopmental outcomes and growth status, were not included. Lastly, the maternal serological status of CMV was not screened and was thus unavailable. Although CMV DNA was detected in 68% of the breast milk samples in our study, it did not accurately reflect maternal seroprevalence.

In conclusion, most preterm infants with pCMV infection *via* breast milk show a favorable clinical course and outcomes, however, serious courses and even lethal outcomes were previously described. Also in our cohort, a high CMV viral load in breast milk was associated with the transmission of the virus to preterm infants, and CMV DNA was detected early (<3 weeks) in the urine in a small (3 of 17) number of infants. Further research is needed to understand the disease burden and establish an effective strategy for the prevention and treatment of severe pCMV infection in extremely preterm or very low birth weight infants.

## Data Availability

The original contributions presented in the study are included in the article/**Supplementary Material**, further inquiries can be directed to the corresponding author/s.
