# Supplementary figures and images for: Clinical presentation and transmission of postnatal cytomegalovirus infection in preterm infants
Source: Front Pediatr. 2022 Nov 21;10:1022869. doi: 10.3389/fped.2022.1022869 (PMC9719915; doi:10.3389/fped.2022.1022869)

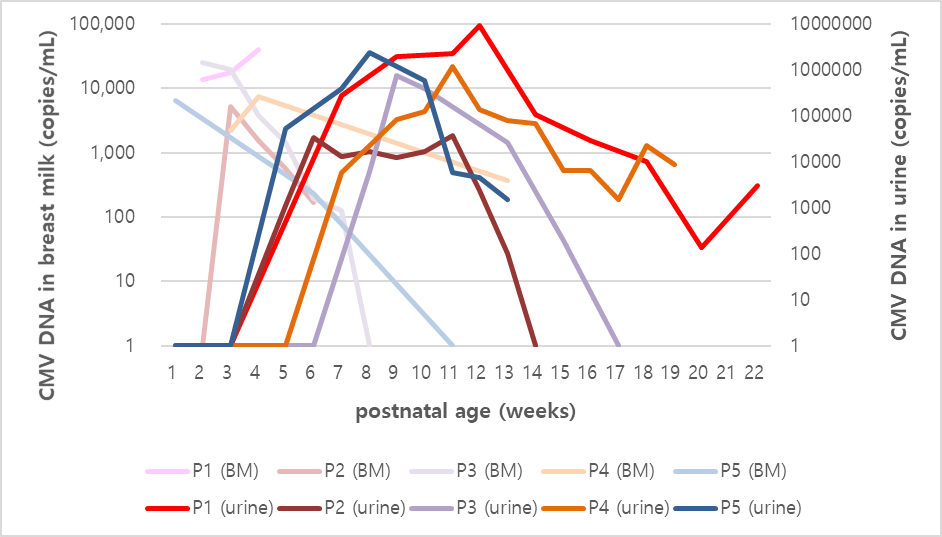

Supplement: Supplementary file 1 [file Image1.tif]
